# Autologous blood pleurodesis: A good choice in patients with persistent air leak

**DOI:** 10.4103/1817-1737.56011

**Published:** 2009

**Authors:** Ufuk Cobanoglu, Mehmet Melek, Yesim Edirne

**Affiliations:** *Department of Chest Surgery, University of Yuzuncu Yil, Turkey*; 1 *Department of Pediatric Surgery, University of Yuzuncu Yil, Turkey*

**Keywords:** Autologous blood, pleurodesis, persistent air leak

## Abstract

**AIM:**

The study compares the efficiency, side effects and complications of autologous blood pleurodesis with talcum powder and tetracycline.

**MATERIALS AND METHODS:**

This prospective study evaluated 50 patients with persistent air leak resulting from primary and secssondary spontaneous pneumothorax between February 2004 and March 2009. The patients inclussded 32 (64.0%) males and 18 (36.0%) females with a median age of 39 years (range 14-69 years). All cases had persistent air leak of more than seven days. Pleurodesis was performed using autologous blood in 20 (40.0%) patients, talc powder in 19 (38.0%) patients and tetracycline in 11 (22.0%) patients through a chest tube. Air leak cessation times after pleurodesis, side effects and pulmonary function tests (PFT) in the first and third months were measured.

**RESULTS:**

Recurrent primary spontaneous pneumothorax was the cause of persistent air leak in all cases. Air leaks were expiratory only in 54.0% of cases. We obtained a success rate of 75.0% using autologous blood, 84.2% using talc powder and 63.6% using tetracycline. Mean air leak termination interval was significantly (*P* < 0.001) shorter in patients treated with autologous blood in comparison to talc powder and tetracycline. We observed a significant (*P* < 0.05) decline in PFT in patients treated with talc powder compared with tetracycline and autologous blood. Vital capacity, FVC and FEV_1_ were significantly lower in patients treated with tetracycline compared with autologous blood.

**CONCLUSION:**

This study shows that autologous blood pleurodesis compared to talc powder and tetracycline is related with shorter leak cessation time and less pulmonary function decline in patients with persistent air leak. We think further randomized clinical trials of pleurodesis as treatment could increase its use in thorax surgery by demonstrating the safety and the efficacy of this procedure.

Persistent air leak is a frequent problem that can occur as a result of traumatic and spontaneous pneumothorax and after pulmonary surgery.[1] This condition causes long durations of hospital stay and carries the risk of respiratory infections, empyema and deep venous thrombosis.[2] Treatment usually includes tube thoracostomy with aspiration and chemical pleurodesis to seal the leak.[3] Other interventions such as re thoracotomy and surgical repair or biological glues are also used.[4] Pleurodesis is caused by adhesion of parietal and visceral pleura to eliminate the pleural space. This can be achieved surgically by thoracotomy or thoracostomy causing mechanical abrasion or by using chemical sclerosing materials via thoracoscopy or chest tube. Several agents such as bleomycine, cyanoacrilate, tetracycline derivates and talc powder are used to perform pleurodesis.[5]

Although several studies on pleurodesis exist, there is no consensus on which agent and what dose should be used. Other authors have used tetracycline derivates and reported about 50% success rates but severe pain after the procedure was observed in some patients. Pleurodesis with talc powder has been used; however, complications such as tachycardia and adult respiratory distress syndrome (ARDS) have also been reported.[6,7]

Pleurodesis with autologous blood has been recommended as an effective, painless, inexpensive and simple method in the treatment of persistent air leaks.[8,9]

This prospective study was conducted to compare the results of pleurodesis performed with autologous blood, talc powder and tetracycline in persistent air leaks.

## Materials and Methods

Between February 2004 and March 2009, 50 patients with persistent air leaks resulting from primary and secondary spontaneous pneumothorax were evaluated. Patients were treated in the clinics of Chest and Pediatric Surgery at the University of Yuzuncu Yil. Patients were selected and included only from primary or secondary pneumothorax cases with persistent air leak of more than seven days. Recurrent pneumothorax cases and persistent pneumothorax cases that underwent an operation were not included. Autologous blood pleurodesis was preferred in relatively young (less than 40 years) cases given the probability of recurrence and surgery. Tetracycline and talc powder were used in patients of the same age group with recurrent pneumothorax and persistent air leak who did not accept surgery. We rated the air leaks from 0 to 4 according to Cerfolio *et al*.[1]

### Pleurodesis

#### Autologous blood

A sample (max. 1 ml/kg) of peripheral blood was taken from the patient and 50 ml of blood was injected immediately. Heparin was not added. No sedation or analgesia was required. All cases underwent this procedure only once.

#### Tetracycline

Tetracycline pleurodesis was performed using 250 mg lidocaine and 20 mg/kg tetracycline in 150 ml saline, as described by Almassi and Haasler.[10]

#### Talc powder

Five grams of sterile talc powder in 40 ml saline and 10 ml prilocaine was introduced into the pleural space for pleurodesis. Patients treated with tetracycline and talc powder required intravenous analgesia in addition.

During the procedures, the chest tube was clamped and disconnected from the water seal; its distal end was cleaned with povidone-iodine and agents were introduced by connecting the cone of the syringe to the tube. Patients rested in contra-lateral decubitus position. The chest tube was rinsed with 10 ml saline solution afterwards and kept 60 cm above the patients' chest for two hours. Patients were allowed to change their positions in bed allowing the agents to distribute in the cavity. The chest tube was connected back to the water seal afterwards.

Presence of air leak for more than 72 hours was accepted as a failure and pleurectomy was performed in these cases with video-assisted thoracoscopic surgery (VATS).

Air leak termination duration and pulmonary function test results (VC, FVC, FEV_1_) of the patients at months 1 and 3 were obtained and analyzed. Spirometry was performed by the Chest Disease Clinic specialists using ZAN100 USB Better Flow (ZAN100) at months 1 and 3 after pleurodesis.

### Analysis

Repeated measurement ANOVA tests were used to investigate the relation between groups regarding differences of VC, FVC and FEV_1_ and to determine significance between means.

One-way ANOVA analysis was used for comparison of air leak termination length between groups. Duncan multiple comparison test was used to determine differences in means between groups. All analysis was performed using SPSS 16.0 software and *P* values of five per cent and one per cent were accepted as significant.

Approval from the Ethic Committee of the University of Yuzuncu Yil was obtained on 24 January 2004 and informed consent from all patients or responsible relatives was also obtained.

## Results

**Figure 1:**
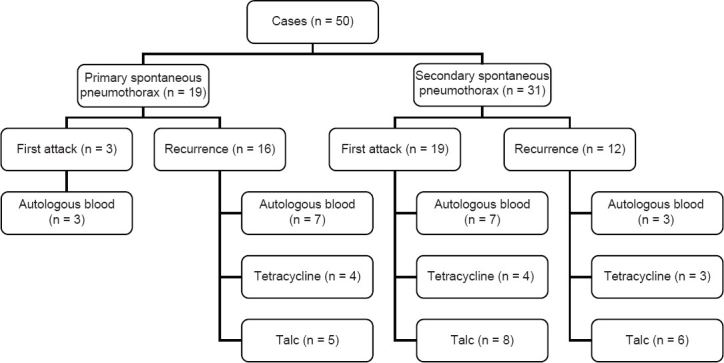
Flow-chart of patients

Leak size was determined as second degree in 62.0% (31) of the cases. The mean duration of air leak was seven to nine days in 48 (96.0%) patients and longer than 10 days in two (4.0%) patients [Table 1]. The cause of persistent air leak was first attack of secondary spontaneous pneumothorax in 19 (38.0%) cases. Distributions of pneumothorax cases are shown in Figure 1. Air leaks in patients treated with autologous blood resolved in the first 12 hours in 20.0% and in the first 24 hours in 35.0%. The air leaks in patients treated with talc powder resolved in the first 48 hours in 36.8% and with tetracycline in 72 hours and beyond in 45.4% of the cases [Table 1]. We obtained a success rate of 75.0% with autologous blood, 84.2% with talc powder and 63.6% with tetracycline use [Table 1].

**Table 1:** Size, duration of air leaks and results of air leak termination times

	Autologous blood	Talc	Tetracycline	Total (%)
Size of air leak*				
0	-	-	-	-
1	5 (41.6)	5 (41.6)	2 (16.8)	12 (24.0)
2	13 (41.9)	11 (35.5)	7 (22.6)	31 (62.0)
3	2 (28.6)	3 (42.8)	2 (28.6)	7 (14.0)
Duration of air leak (days)				
7	7 (36.8)	8 (42.1)	4 (21.1)	19 (38.0)
8	9 (40.9)	8 (36.4)	5 (22.7)	22 (44.0)
9	3 (42.8)	2 (28.6)	2 (28.6)	7 (14.0)
>10	1 (50)	1 (50)	-	2 (4.0)
Air leak termination times in hours (n = 38)				
12	4 (20.0)	2 (10.0)	-	6 (15.8)
24	7 (35.0)	5 (26.4)	-	12 (31.6)
48	2 (10)	7 (36.8)	2 (18.2)	11 (28.9)
>72	2 (10)	2 (10)	5 (45.4)	9 (23.7)
Successful procedure	15 (75.0)	16 (84.2)	7 (63.6)	38 (76.0)
Failure	5 (25.0)	3 (15.8)	4 (36.4)	12 (24.0)

* : 0 = No air leak, 1 = forced expiratory, 2 = expiratory only, 3 = inspiratory only, 4 = continuous

Air leak cessation times were significantly shorter after autologous blood injections in comparison to talc powder and tetracycline use (*P* < 0.001). There was no significant difference in leak cessation time between talc powder and tetracycline (*P* > 0.05) [Table 2].

**Table 2:** Comparison of air leak termination times

	n	Mean (hrs)	SD	SEM	Min. (hrs)	Max. (hrs)	*P*
Autologous blood	20	27.2	17.87	5.38	12	72	0.000
Talc	19	51.0	15.38	5.43	24	72	
Tetracycline	11	64.0	12.39	5.06	48	72	
Total	50	43.6	21.87	4.37	12	72	

SD = standard deviation, SEM = standard error of mean

Different letters resemble statistical difference between means (*P* < 0.001).

We performed pleurectomy and pleural abrasion with VATS in 12 (24.0%) patients with failure to stop the leak after pleurodesis. While no side effects seemed to occur in patients treated with autologous blood pleurodesis, fever (63.2%) and dyspnea (57.9%) with talc slurry and pain (90.9%) with tetracycline were observed most of the time [Table 3].

**Table 3:** Side effects and complications of pleurodesing agents

	Autologous blood	Talc powder	Tetracycline
Side effects	-	Fever (n = 12)	Fever (n = 9)
		Pain (n = 8)	Pain (n = 10)
		Emesis-vomiting (n = 4)	Dyspnea (n = 4)
		Hypotension (n = 3)	Elevated liver
		Supraventricular	enzymes (n = 5)
		tachycardia (n = 1)	
		Convulsion (n = 1)	
		Dyspnea (n = 11)	
Complications	Empyema (n = 1)	ARDS (n = 1)	Empyema (n = 1)

ARDS = Adult respiratory distress syndrome

Empyema after tetracycline pleurodesis was observed in one case at day 15. The same complication was observed in one patient on ninth day after blood pleurodesis. In both cases, microbiological analysis of the pleural fluid revealed Staphylococcus epidermidis. Both patients received appropriate antibiotics and irrigation with antiseptically fluids to the pleural space was done. Although empyema resolved in both cases, the leaks persist and pleurectomy with VATS was performed.

One patient developed dyspnea with hypoxia and hypercarbia two hours after talc powder treatment. Mechanical ventilation for two days resulted in improved respiratory functions and oxygenation and the patient was weaned from the ventilator.

Three patients (9.67%) treated with tetracycline developed recurrent pneumothorax and pleurectomy and pleural abrasion was performed with VATS.

PFT were performed in all patients at first and third month by spirometry. Vital Capacity, FVC and FEV_1_ were significantly lower in patients treated with tetracycline compared with autologous blood use, and significantly lower in patients treated with talc powder compared with autologous blood and tetracycline use (*P* < 0.05) [Table 4].

**Table 4:** Pulmonary function test results

		Autologous blood			Talc powder			Tetracycline		
				
		Mean	SD	Range	Mean	SD	Range	Mean	SD	Range
VC	1 mo	2.73	0.20	2.44-3.11	2.03	0.15	1.81-2.32	2.43	0.30	2.11-2.87
	3 mo	2.79	0.20	2.41-3.10	2.05	0.12	1.87-2.32	2.61	0.22	2.31-2.98
FVC	1 mo	2.68	0.22	2.37-3.11	1.68	0.14	1.56-1.98	1.93	0.15	1.79-2.28
	3 mo	2.81	0.18	2.61-3.07	1.99	0.09	1.87-2.17	2.32	0.26	1.21-2.71
FEV_1_	1 mo	2.73	0.17	2.37-2.86	1.61	0.14	1.46-1.87	2.42	0.23	1.87-2.74
	3 mo	2.77	0.13	2.56-2.94	1.92	0.09	1.77-2.07	2.41	0.20	2.21-2.77

Different lower case letters in the same rows represent significant difference between groups (*P* < 0.05).

Different upper case letters in the same columns represent significant difference between groups (*P* < 0.05).

SD = standard deviation, VC = vital capacity, FVC = forced vital capacity, FEV_1_ = forced expiratory volume in 1 second.

## Discussion

Persistent air leak is a common complication after thorax surgery.[1] While Rice and Kirby have reported a 15.2% rate of air leak persisting more than seven days after pulmonary lobectomy in 197 consecutive patients,[2] air leak was observed in 27 (14.8%) of 182 patients treated by VATS wedge resection for spontaneous pneumothorax in another study.[11]

Our patients consisted of secondary or primary spontaneous pneumothorax cases. No technique has proven superiority to others for the treatment of persistent air leaks. Some of the procedures applied by surgeons are; a drain *in situ* and a Heimlich valve,[3] more aggressive approaches such as intra- pleural chemical agents (pleurodesis) or even primary repair by re-operation and injection of fibrin glue.[4]

The aim of pleurodesis is to achieve pleural attachment.[12] Therefore, expansion of the lungs and repair of pneumothorax before the procedure are mandatory. Pleurodesis is possible with agents such as tetracycline, talc powder and autologous blood.[8] Tetracycline is an antibiotic commonly used for its sclerosing effect. According to Macoviak *et al*., tetracycline only produces an inflammatory reaction and scarring but no “patch” effect.[12] Its efficiency is reported to be more or less 50%.[7,8] Talc powder pleurodesis is probably effective through interleukin mediated polymorphonuclear neutrophil migration and monocyte infiltration with inflammation as a result. The risk of mesothelioma is minimized with the elimination of asbestos from talc powder.[13]

On the other hand, restrictive respiratory distress has developed after talc powder treatment in 75 patients in the long-term in a study.[7,13] In addition, sudden respiratory distress and death has been reported following talc powder use.[13,14]

Pleurodesis with autologous blood was first performed by Robinson for treating patients with persistent air leaks due to spontaneous pneumothorax.[15] Autologous blood pleurodesis could involve two factors working together: The blockage of a small air leak by forming a clot and the fibrogenic activity of the blood in the pleural cavity producing inflammation and irritation of both pleurae.[12,15] Tetracycline and talc powder induce probably only inflammation and scarring, with no “patch” effect.

Autologous blood pleurodesis for the treatment of persistent air leaks, especially in patients with spontaneous pneumothorax, has been in use since 1992.[9,16] In these studies the amount of autologous blood ranged between 50 to 250 ml as daily 50 ml injection repeated until success. We used only one single injection of 50 ml of blood in all our cases and obtained the desired effect also described by Dumire *et al*.[8] and Cagirici *et al*.[16] The reasons for not injecting more than 50 ml of blood included the concerns about injecting an ideal medium for bacteria in the pleural space in addition to the increased risk of bacterial contamination due to repeated manipulation of the drains.

In available literature, the time between the operation and the blood patch pleurodesis has varied from 10 to 23 days as seen in the series reported by Rivas de Andres,[3] but has been up to five weeks.[9] In all our cases we preferred to perform pleurodesis after the seventh day despite the existing discussions on the accurate time. Tetracycline induces only inflammation in the pleural cavity resulting in adhesion; air leak cessation is not expected before 3 to 5 days.[12] We observed similar results in our study; air leak cessation time exceeded 48 hours in average with tetracycline use. A study reported air leak cessation in the first 12 hours in 72.7% of cases and cessation of all leaks in 48 hours using autologous blood[17] while in two other series this period was under 24 hours.[3,8] In our study, air leak cessation time was under 24 hours in the majority of the cases that underwent autologous blood pleurodesis and this seems to be the result of the “patch” effect. Success rates of 50-72% for tetracycline and 85-95% for talc pleurodesis have been reported in literature.[6,7,14,18] However, success rates of 59-100% are reported with autologous blood pleurodesis adding that it is a simple, inexpensive and safe procedure. Success is even approved for cases with unexpanded lungs.[8,9,15,16]

We achieved a success rate of 75.0% with autologous blood which was close to the success rate of talc powder (84.2%) but exceeding tetracycline (63.6%). De Vires and Wolfe[19] reported persistent air leaks in 32% and Granke *et al*.[20] in 22.4% of cases treated with a chest drain only. These high recurrences emphasize the necessity of pleurodesis in the treatment of persistent air leaks. Tanaka[21] reported persistence in 18.8% patients treated with tetracycline while Jantzing[22] reported persistence in 4% with quinacrine use. Our failure rates were 25.0%, 15.8% and 36.4% respectively for autologous blood, talc powder and tetracycline. The rate of recurrence at the ipsilateral side was reported higher (50%) with tetracycline use compared with the rates reported in literature. Reasons for failure in pleurodesis could be working with suboptimal techniques or inaccurate patients. Options in these cases are re performing sclerosing agents through the chest tube, thoracoscopic talc use or pleurectomy. We have chosen pleurectomy in our cases and no recurrence was observed.

Possible complications of talc and tetracycline are pleural thickening, diffuse fibrosis and decline in pulmonary functions. [13,22] Restrictive respiratory insufficiency has been reported with talc use in the long term.[6,13] We have observed serious decline in the PFT results of patients treated with tetracycline and talc powder compared with autologous blood that was statistically significant.

Pleurodesis with tetracycline can cause severe pain.[8] Therefore, intrapleural lidocaine, in addition to intravenous sedation, is required in these patients while no anesthesia is necessary with autologous blood use. Pain was the most frequent side effect in our patients treated with tetracycline.

Side effects due to talc powder can be caused by the systemic reflection of severe inflammation in the pleural space. Dose related life-threatening side effects are also reported. Higher doses of talc seem to induce ARDS as reported by Antunes and Neville, which have used 10 g of talc.[23] We have used five grams of talc in our patients and this could be the reason of no severe side effects except in one patient.

Chest pain and fever are the most frequent side effects of pleurodesing agents.[17,20] Fever and chest pain which resolved in 24 hours with paracetamol use were observed in our patients

Life-threatening complications can occur after talc pleurodesis. [7,14,18] We observed supraventricular tachycardia after talc pleurodesis in one patient that could not linked to an existing pathology but it resolved spontaneously.

Fever, pleural effusion and empyema are reported with autologous blood pleurodesis.[15–17] Robinson found pleural infection in four per cent of patients treated with autologous blood in his study.[15] We preferred to use a single injection of 50 ml of blood to prevent the accumulation of contaminated bacteria necessary for infecting the pleural space. Strict aseptic conditions were accomplished during the procedures. An infection occurred only in one patient in the form of empyema that resolved under antibiotherapy.

Obstruction of the catheter is an important problem which occurs during autologous blood pleurodesis. Thin catheters help obtain blood from the vena slowly and with a thin syringe and delay in introducing can end with obstruction resulting in tension pneumothorax.[9] Therefore, the use of big sized chest tubes and syringes of 18 gauge and 0.9 mm for peripheral blood samples are recommended. Flushing the tube after the procedure with normal saline is also suggested.[8,15] We accomplished all suggestions in literature and tension pneumothorax was not observed in any of our patients.

## Conclusion

We conclude that pleurodesis with autologous blood is an acceptable, painless, inexpensive and simple method in the treatment of recurrent primary spontaneous pneumothorax. We demonstrated that autologous blood is faster in ceasing air leaks when compared with talc powder and tetracycline. Its effectiveness is comparable to talc powder but superior to tetracycline with fewer side effects in comparison to both agents. The reason why this procedure is neglected could be the small number of cases and reports published. Further randomized clinical studies are needed to extend the use of autologous blood pleurodesis.
